# Case Report: Rare Bilateral Double Profunda Femoris Artery Variation: Clinical Implications and Considerations

**DOI:** 10.12688/f1000research.151518.3

**Published:** 2025-09-03

**Authors:** Prasanna Gaydhanker, Soumya Vinod, Ernest Adeghate, Sahar Mohsin

**Affiliations:** 1 Anatomy, United Arab Emirates University, Al Ain, Abu Dhabi, 15551, United Arab Emirates

**Keywords:** Profunda femoral artery, Anatomical variation, Cadaver, Femoral artery, Bilateral, Nerve, Complications, Surgeons.

## Abstract

**Introduction:**

The profunda femoris artery (PFA) is the largest branch of the femoral artery supplying the thigh. The study reports a rare occurrence of bilateral double profunda artery in a male cadaver, aiming to inform surgeons of such variations to avoid complications during surgical interventions.

**Methods:**

This study was conducted during routine dissections using 5-10% formalin-fixed lower limbs of a male cadaver at the Department of Anatomy, College of Medicine and Health Sciences, United Arab Emirates University, UAE

**Results:**

In the right lower limb, two profunda femoris artery (PFA) branches arise from the common femoral artery (CFA) 5 cm from the inguinal ligament, one laterally and one posteromedially. The medial (MCFA) and lateral circumflex (LCFA) arteries branch from these PFAs respectively. The femoral nerve is anterior to the lateral PFA and LCFA and lateral to the posteromedial PFA. The obturator nerve runs posterior to the posteromedial PFA. In the left lower limb, two PFAs originate from the CFA, 2.5 and 5 cm from the inguinal ligament, lateral and posterior to the CFA, respectively. The LCFA stems from the upper lateral branch of the duplicated PFA, while the medial MCFA arises from the CFA. The femoral nerve lies anterior to both the PFA and LCFA. The obturator nerve is posterior to the MCFA. The femoral vein crosses over the MCFA and the lower posterior PFA, distal to the MCFA origin.

**Conclusion:**

This report enriches understanding of PFA anatomy and its variations, emphasizing the importance of tailored surgical approaches to minimize risks and ensure procedure success.

## Background

The thigh region of the lower limb is supplied by the femoral artery and its branches. The femoral artery is the continuation of the external iliac artery distal to the inguinal ligament, which is located approximately halfway between the anterior superior iliac spine and the pubic symphysis. The common iliac artery divides into the internal and external iliac arteries at the level of L5–S1. Positioned anteriorly within the femoral triangle of the thigh, the femoral artery passes through an opening in the adductor magnus muscle before becoming the popliteal artery in the area behind the knee joint. It then gives off branches to supply blood to the leg and foot regions. ^1^

This study explores the variability observed in the primary branch emanating from the femoral artery in the thigh region, the profunda femoris artery (PFA). The PFA supplies the medial and posterior compartments in the thigh region. Both the femoral artery and PFA play crucial roles in various clinical contexts, ranging from diagnostic imaging to surgical reconstruction and vascular access. ^2^ Therefore, awareness of variations in PFA branches and their relationship with nerves enhances surgical precision, reduces complications, and ultimately improves patient outcomes across various clinical specialties, including surgery, interventional radiology, and neurology.

The PFA originates from the femoral artery approximately 3.5 cm below the inguinal ligament. ^1^ The PFA is also referred to as the ‘deep femoral artery’ and, in this case, the segment of the femoral artery preceding the origin of the PFA is referred to as the common femoral artery (CFA), while the portion of the femoral artery following the origin of the PFA is termed the ‘superficial femoral artery’ (SFA). The PFA at its origin is lateral and then spirals posterior to the femoral artery. Proximally, the femoral vein lies medially and the PFA courses between the pectineus and the adductor longus muscles, subsequently passing between the adductor longus and brevis muscles, and finally descends in a gap bordered by the adductor longus and adductor magnus muscles. It concludes by transitioning into a fourth perforating artery and establishing connections with the muscular branches of the popliteal artery within the adductor magnus muscle. ^1^

Multiple variations have been documented regarding the profunda femoris artery (PFA), including alternative origins such as medial, posterior, or posterolateral instead of the typical lateral position, as well as instances where it shares a common origin with one of the circumflex arteries arising from the femoral artery. ^3– 6^ The superficial branches of the femoral artery are also reported to be arising from the PFA. However, duplication of the PFA is uncommon with a reported prevalence of <1%, ^7, 8^ Tsoucalas et al., 2018 reported a double PFA in the left thigh of a female cadaver, no variations were found in the distribution pattern of each branch, and the relationship with nerves was not discussed. ^7^

The PFA emits two circumflex arteries, medial and lateral, as well as perforating arteries through which it delivers oxygenated nutrients to the proximal aspect of the femur and the ‘medial’ and ‘posterior’ compartments of the thigh, aiding in adduction, extension, and flexion movements. ^1– 3^

Anatomists have observed variations in the origin of the circumflex arteries as well. The lateral circumflex artery (LCFA) is the major branch of the PFA found in 67% of the population originating 1.5 cm below the lateral aspect of the PFA, however, in 20% of people may arise directly from the femoral artery. ^9^ The LCFA travels horizontally between the divisions of the femoral nerve and posteriorly passes behind the sartorius and rectus femoris muscles. ^3^ Afterwards, it splits into ascending, transverse, and descending branches. Typically, the artery runs anteriorly to the femoral neck. ^2^ Variations in the relationship of the femoral nerve to the PFA and circumflex arteries are also reported. ^1, 10– 12^

The medial circumflex artery (MCFA) is important for the blood supply to the proximal region of the femur. It is given off from the PFA posteriorly, anterior to the iliopsoas and behind the pectineus muscles. It can be traced further between the obturator externus and the adductor brevis muscles. Occasionally in 25% of the population it may originate directly from the femoral artery. Damage to the MCFA following a femoral neck fracture can lead to avascular necrosis of the femoral neck or head. ^1, 12^

**Aim & Objectives**: Here we report a case of bilateral occurrence of a double PFA originating from the femoral artery in an adult male cadaver and its relationship with nerves. Data regarding arterial variations is significant for orthopedic surgeons, vascular specialists, and radiologists as it aids in mitigating complications during surgical procedures. ^3^ Knowledge of the relationship between the PFA and nerves helps minimize the risk of iatrogenic nerve injury during surgical procedures which can significantly impact patient outcomes and quality of life.

## Methods

This case report is based on the dissection of the lower limbs of a single male cadaver, which had been fixed in the 5-10% formalin (Manf-Cibachem, Cat No: 233344). The cadaver was obtained from Body Scientific International, LLC, through Medcure and the Anatomical Gift Association of Illinois as part of a body donation program for teaching and research purposes. The study was conducted in the ‘Department of Anatomy, College of Medicine and Health Sciences, United Arab Emirates University, UAE’ during standard dissection for undergraduates as per the ‘Cunninghams Manual of Practical Anatomy’ instructions. ^13^ The dissections involved examining the thigh compartments and tracing the femoral artery and its branches from beginning to end. The origin, path, and branching pattern of the femoral artery were explored on both sides, and images were taken and compared with previous literature.

## Results

### Origin of double profunda femoral arteries in the right thigh region of the lower limb

**Figure 1.**
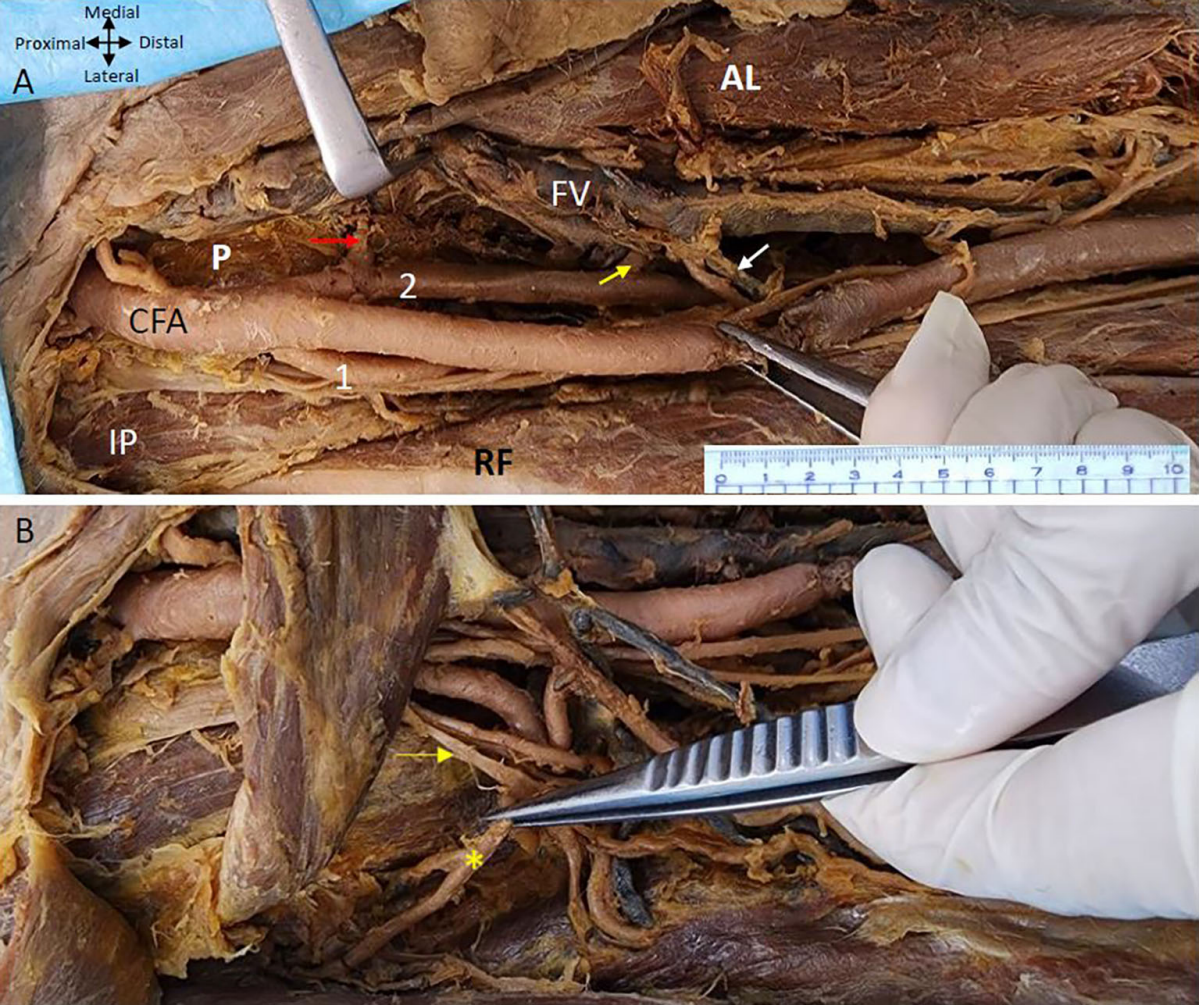
Double profunda artery in the right thigh region. (A) Double Profunda femoris artery (PFA) origin from the common femoral artery (CFA). The lateral branch is labelled as (1) and posteromedial one by (2). Muscular branches from the posteromedial PFA to the adductor muscles ae indicated by yellow arrow. Adductor longus, rectus femoris, pectineus and iliopsoas muscles are indicated by (AL), (RF) (P), and (IP) respectively. The Middle Circumflex Femoral artery (MCFA) originating from the posteromedial PFA is shown by a red arrow. The femoral vein (FV) lies medial to the femoral artery and its branches. Tributaries of the femoral vein in anterior relation to one of the branches of the posteromedial PFA are shown by a white arrow (B) The rectus femoris (RF) muscle is reflected to show the lateral circumflex femoral artery (LCFA) coming from the lateral PFA in the right lower limb. The LCFA is shown by a yellow asterisk (*) coming off from the lateral PFA posterior to the branches of the femoral nerve which is shown by the yellow arrow.

It was observed that the femoral artery follows the usual course passes under the inguinal ligament enters the femoral triangle bounded medially by the adductor longus and laterally by the sartorius with the femoral vein lying medially and ‘femoral nerve laterally’. The variation is noticed in the branching pattern of CFA as it was giving two profunda arteries bilaterally. In the right lower limb both PFA are arising from the CFA at the same level 5 cm from the inguinal ligament. However, one is arising laterally and the other is coming off posteromedially from the CFA ( Figure 1). The lateral profunda femoral artery is bounded laterally by the iliopsoas muscle and the posteromedial branch traverses between the pectineus and the adductor longus muscle.

**Figure 2.**
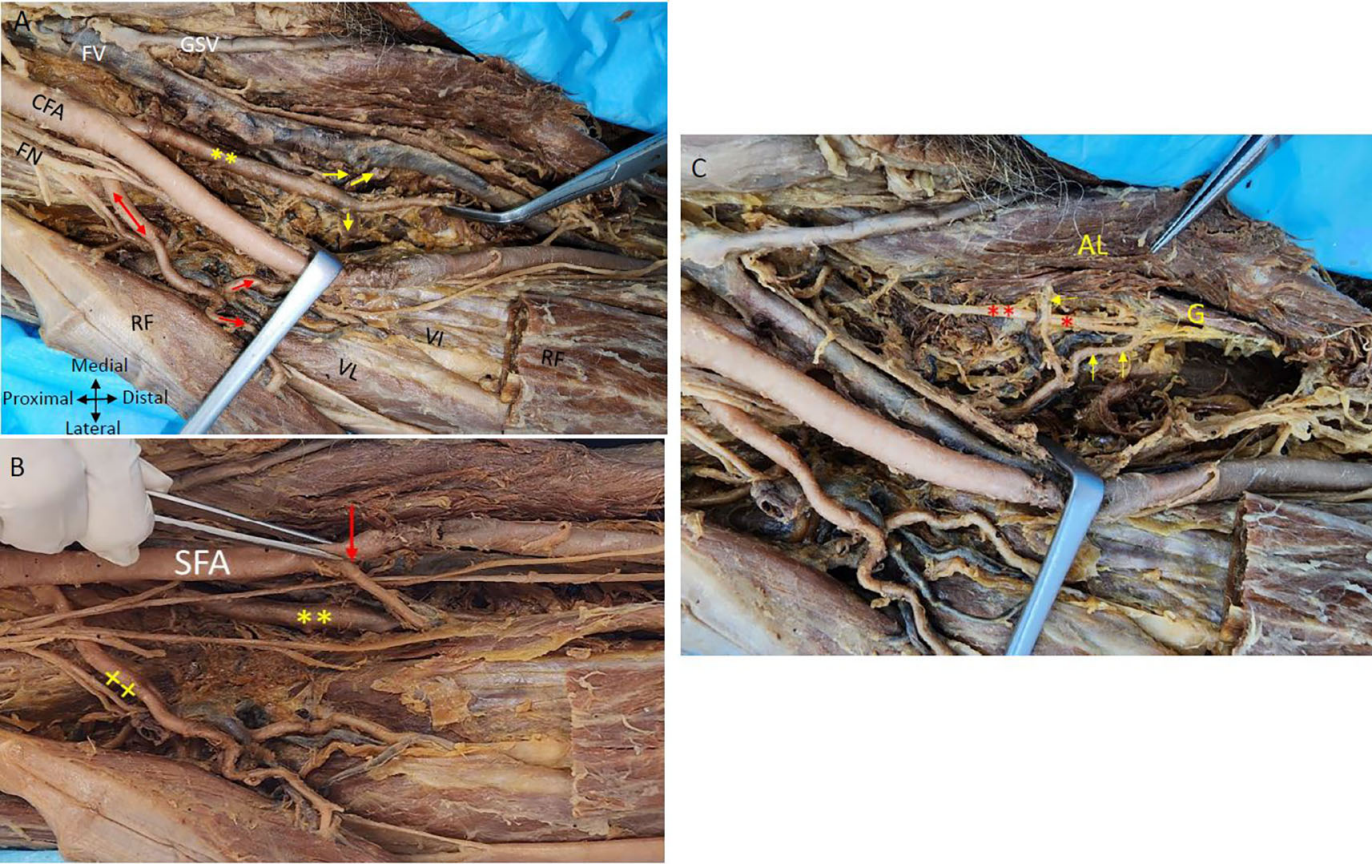
Relationship of nerves and veins to the profunda arteries in the right thigh region. (A) Showing double profunda arteries arising from the CFA in the right thigh region. The femoral nerve (FN) is lateral to the CFA and some branches of FN are related anteriorly to the lateral PFA (red double-head arrow). The femoral vein (FV) is medial to the femoral artery and the great saphenous vein (GSV). The posteromedial profunda femoris branch shown by the yellow (**) gives multiple branches to the adductor muscles (yellow arrows). The lateral branch gives muscular branches indicated by red arrows to the quadriceps femoris (rectus femoris (RF); vastus lateralis (VL) and vastus intermedialis (VI). Adductor Longus and rectus femoris muscles are indicated by (AL) and (RF). The rectus femoris is cut and reflected to show the vasti muscles (B) The CFA gives out two profunda femorals and continues as superficial femoral artery (SFA). One arising posteromedially (**) and another profunda femoral coming off laterally (xx). The rectus femoris muscle is cut and reflected to see the branches of the PFA. The lateral branch is giving branches to the quadriceps femoris shown (x). The vastus medialis muscle is getting a direct branch from the superficial femoral artery indicated by the red arrow in right thigh region (C) The branches from the posteromedial profunda to the adductor longus muscle (AL) and the gracilis muscle (G) are shown by yellow arrows are related to the branches of the obturator nerve red (*) in the right thigh region.

The posteromedial branch of the PFA gives muscular branches to the adductor muscles ( Figures 1 & 2). The posteromedial branch in the mid-thigh gives multiple branches to the adductor magnus muscle as shown in Figure 2A. The femoral vein lies medial to the posteromedial branch of the PFA ( Figures 1 & 2). The posteromedial profunda artery goes from posteromedial to posterolateral in mid-thigh running behind the SFA ( Figure 1). The vastus medialis muscle receiving a branch directly from the SFA is shown in Figure 2B.

The femoral nerve is anterior to the lateral profunda and lateral to the posteromedial PFA in the right thigh region. The lateral PFA laterally traversing between the divisions of the femoral nerve gives branches to the quadriceps femoris muscle. An LCFA is coming off the lateral PFA posterior to the branches of the femoral nerve ( Figure 2B). The obturator nerve is positioned posterior to the branches originating from the posteromedial PFA towards the adductor longus muscle. The posteromedial PFA gives a tributary to the gracilis muscle, initially positioned lateral to the ‘obturator nerve’ and subsequently coursing between its branches that supply the gracilis muscle ( Figure 2C).

The MCFA that supplies the neck of the femur can be seen coming off the posteromedial PFA and traversing medially anterior to the pectineus muscle to supply the neck of the femur ( Figure 1A). The branches of the posteromedial branch of the profunda femoris are related closely to the tributaries of the femoral vein some of them crossing anterior to the branches of the artery ( Figure 1A). The LCFA comes off from the lateral PFA posterior to the branches of the femoral nerve ( Figure 1B).

### Origin of double profunda femoral arteries in the left thigh region of the lower limb

**Figure 3.**
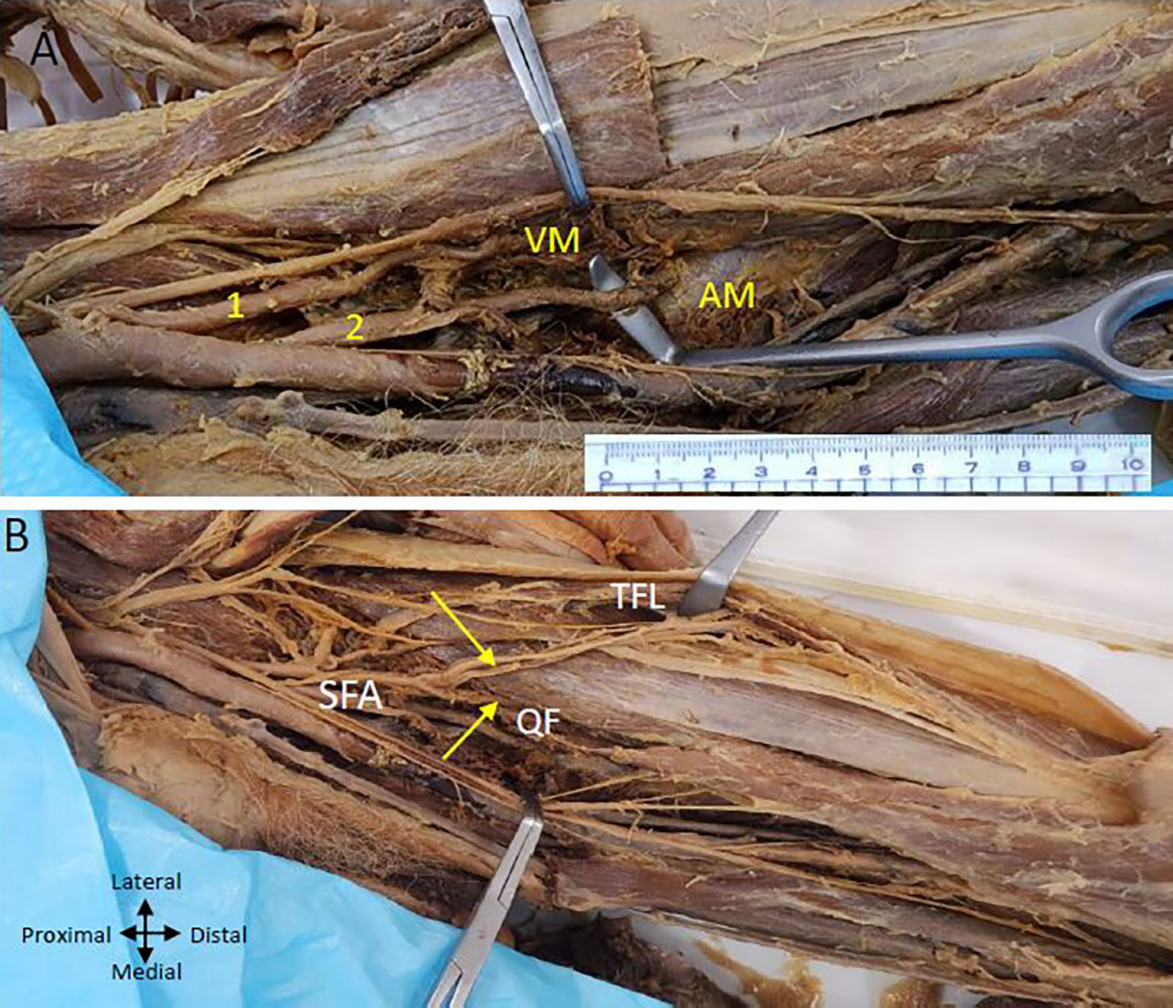
Double profunda artery in the left thigh region. (A) Upper (lateral) profunda (1) piercing the vastus medialis (VM muscle and lower (2) (posterolateral) one pierces the adductor magnus (AM) muscle and Superficial Femoral artery (SFA) is passing through the adductor hiatus (B) Upper profunda giving branch to tensor fasciae latae muscle (TFL) and quadriceps femoris (QF) shown by yellow arrows.

On the left lower limb, we also found two profunda femoris arteries arising from the CFA ( Figure 3). The first PFA commences laterally from the CFA femoral artery, approximately 2.5 centimetres from the inguinal ligament, while the second PFA originates below the first one at a distance of 5 centimetres from the inguinal ligament and is posterior to the CFA at its commencement and later traverses laterally to the femoral artery. The lower posterior PFA is bigger than the upper lateral PFA.

The upper lateral profunda supplies the quadriceps femoris muscle ( Figure 3) and ends by piercing the vastus medialis muscle. The upper branch also gives branches to the tensor fasciae latae muscle on the outer aspect of the thigh ( Figure 3B). A lower posterior profunda gives perforating arteries to the adductor muscles and ends by piercing the adductor magnus muscle above the adductor hiatus ( Figure 3A & B).

**Figure 4.**
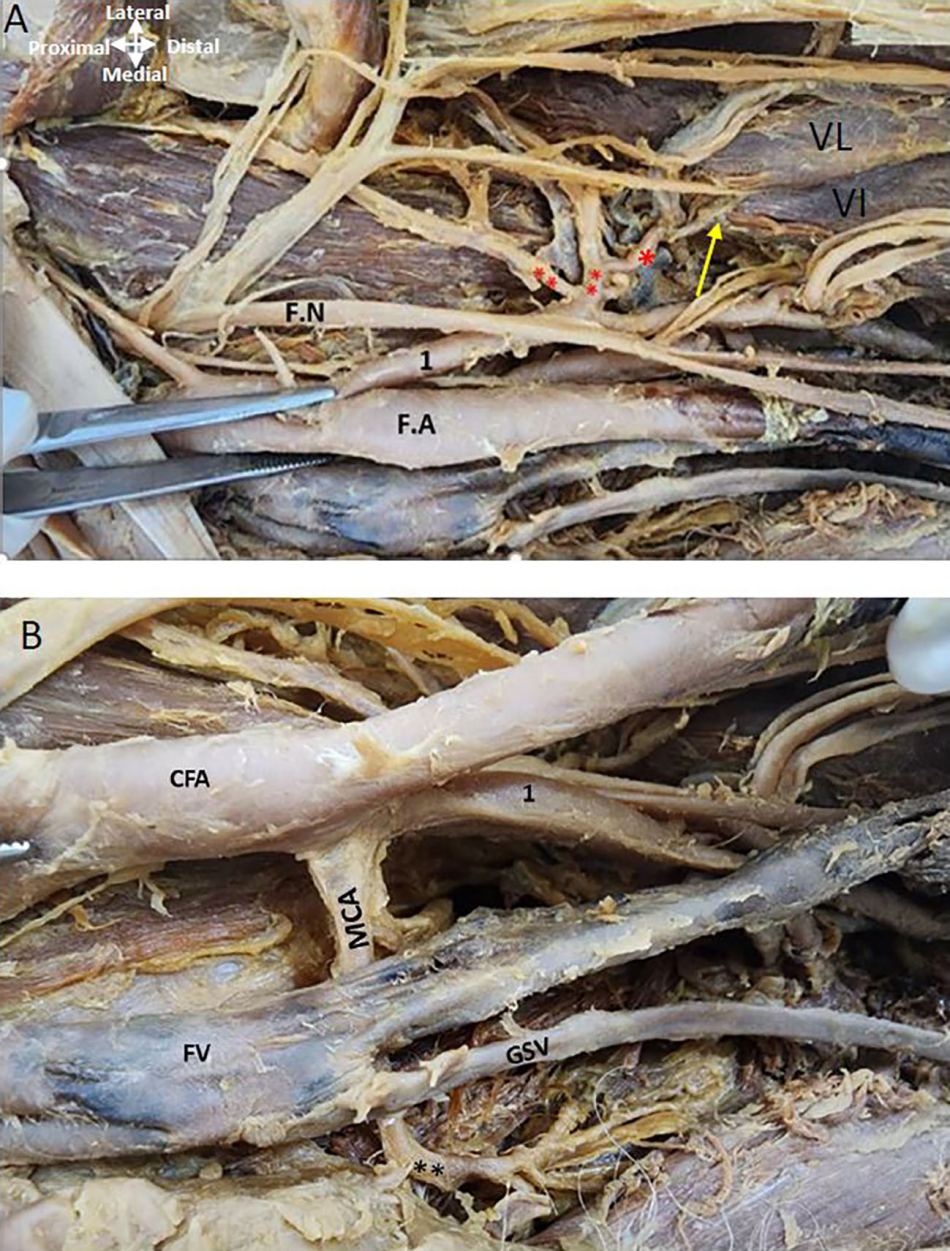
Origin of circumflex arteries in the left thigh region. (A) Upper lateral profunda labelled as 1 is giving out lateral circumflex artery (**) posterior to the femoral nerve (F.N) and its branches. Ascending, transverse, and descending branches from the lateral circumflex are shown (**). The descending branch is shown by the yellow arrow from LCFA is given to the vastus lateralis (VL) and vastus intermedialis (VI). (B) The common femoral artery (CFA) is giving out lower profunda artery labelled as (1) and medial circumflex artery (MCA) is originating from the FA posterior to the femoral vein (FV) in the left thigh region. The great saphenous vein (GSV) opens into the femoral vein.

The LCFA is given off from the upper lateral profunda artery vessels in the left thigh region ( Figure 4A). The LCFA vessel was found to pass posterior to the sartorius and rectus femoris muscles and is crossed by the femoral nerve and its branches. Transverse, ascending, and descending branches from the lateral circumflex artery are depicted in Figure 4, directed towards the vasti muscles. The MCFA however originates from the femoral artery slightly above the lower profunda artery, as illustrated in Figure 4B.

**Figure 5.**
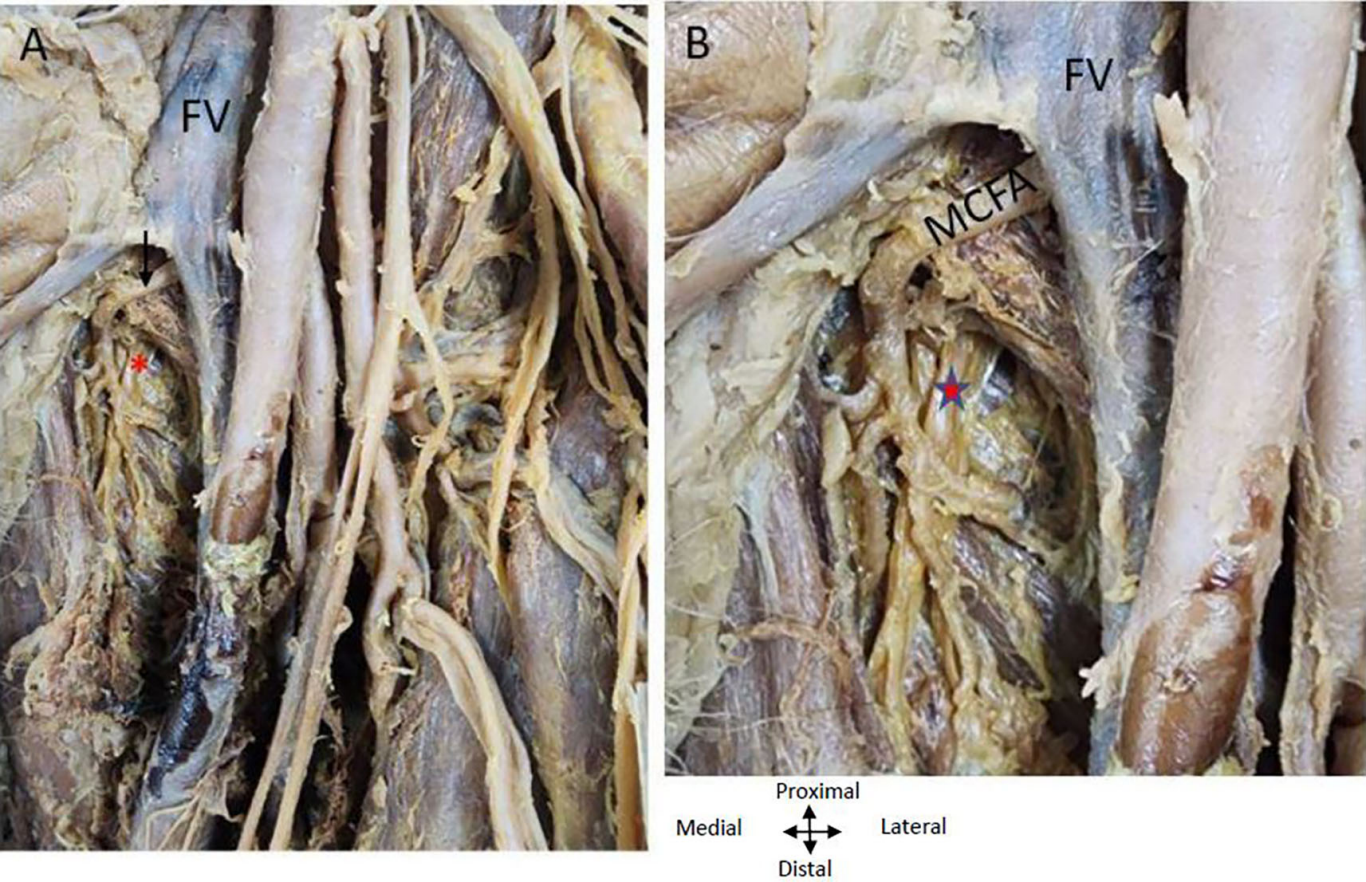
Relationship of nerves and vein to the MCFA in the left thigh region. (A) Obturator nerve is shown by red asterisk is posterior to the medial circumflex femoral A (MCFA) shown by an arrow. Femoral nerve (FN) crossing over the PFA (B) Magnified image showing MCFA crossing over the obturator nerve. Femoral vein (FV) is anterior to the MCFA in the left thigh region.

The femoral nerve lies lateral to the femoral artery proximally and distally it is related anteriorly to both profunda vessels ( Figure 5A). The obturator nerve is posterior to the MCFA ( Figure 5B).

### Relationship of the profunda arteries to the femoral vein in the right and left thigh region of the lower limb

It was found that as the femoral vein ascends through the adductor canal, it follows a standard trajectory, initially situated posterolateral to the femoral artery. Subsequently, it shifts to a posterior position before assuming a medial placement within the femoral triangle, where it consistently lies medially to the femoral artery at its commencement in both limbs ( Figures 1A, 4B, 5). In the left thigh region, the ‘femoral vein’ crosses over the ‘medial circumflex femoral artery’ (MCFA) and is found anterior to the lower posterior PFA, distal to the origin of the MCFA before the lower PFA perforates the adductor muscles (see Figure 4B and Figure 5).

## Discussion

Blood vessel formation involves vasculogenesis and angiogenesis. ^14^ ^–^ ^16^ The molecular control of vascular development is complex, involving growth factors and inhibitors. Vascular remodeling requires vessel sprouting, intussusceptive growth, and regulation by hemodynamic, growth, and inhibitory factors, along with the tissue environment. Variations in these processes, due to genetic factors, failed regulation, or environmental influences (e.g., hypoxemia), can lead to vessel abnormalities like duplication, hypoplasia, or aplasia. ^17^ ^–^ ^19^

The common dorsal aorta forms intersegmental arteries, with the external iliac artery developing from the fifth lumbar intersegmental artery. ^20^ The primary (axial) artery of the lower limb called the sciatic artery, emerges from the common iliac artery and develops from the umbilical artery of the dorsal aorta. ^14^

It extends from the medial thigh to the lateral leg and forms a plantar plexus in the sole. The external iliac/femoral artery penetrates into the lower limb after the axial artery, and it transiently joins the axial/sciatic artery near the knee to form the popliteal artery. ^20^ Between the 6 mm and 33 mm stages, the sciatic artery regresses and the dorsal thigh is primarily supplied by the profunda femoris, which branches off the femoral artery. ^21^ ^,^ ^22^ However, persistence of the sciatic artery as the principal blood supply to the lower limb in adulthood is an uncommon vascular abnormality with potential surgical implications. ^23^ The arterial system of the lower limb is fully formed by the 33-mm stage of embryonic development. The embryonic femoral artery at first comprises several vascular channels, which gradually merge to give rise to the profunda femoral arteries. If these channels fail to fuse properly, a duplicated profunda femoris artery may result. ^24^ The vasculature adopts its adult configuration by the 8th week of embryonic development. Other rare variations include an absent deep femoral artery and lateral and medial circumflex arteries originating from the common or superficial femoral artery instead of the deep femoral artery. ^25^ ^,^ ^26^

Anatomical variations of the femoral artery are uncommon and may include the absence of the profunda femoris artery, a persistent sciatic artery, or duplication of the superficial femoral artery. Most of these variations result from the persistence of primitive arterial segments, abnormal fusions, or segmental hypoplasia or aplasia. ^15^ ^,^ ^19^

The MCFA plays a crucial role in the vascularization of the head and neck of the femur, as indicated by previous research. ^26, 27^ There is a discrepancy in the literature regarding the origin of circumflex arteries and their relationship to the anatomical structures, their origin has been documented to occur from the medial or posterior aspect of the ‘deep femoral artery’. ^1^ Understanding such variations is important to prevent iatrogenic errors in various surgical procedures. ^10, 28^

A prior study involving 342 dissected hemipelves, reported diverse origins of the MCFA. ^26^ It arose from the ‘common’ and ‘deep femoral’ artery in 39.3%, from the superficial femoral artery in 2.5%, and the LCFA in 0.6%. Notably, it was found to be congenitally absent in 0.6%. These findings underscore the variability in the anatomy of the MCFA, which holds clinical significance for surgical interventions.

The presence of bilaterally arising double profunda femoris arteries from the CFA is a noteworthy anatomical variation with crucial implications for medical professionals. This variation can pose challenges during vascular procedures such as, bypass grafting, and endovascular interventions, where an unexpected arterial branching pattern may increase the risk of inadvertent injury, misidentification, or inadequate perfusion. In vascular surgery, the presence of a second PFA could be overlooked during angiography, potentially leading to failed bypass grafts, incomplete aneurysm embolization or repair, or misinterpretation of bleeding sources. In orthopedic and trauma surgery, particularly in cases of pelvic or femoral fractures, attempts to control hemorrhage could fail if only one of two major vessels is ligated or embolized. Similarly, in reconstructive surgery, unrecognized variations may compromise the viability of essential tissue flaps that rely on perforators from the PFA system, such as the anterolateral thigh (ALT) flap, by creating an unpredictable blood supply. Understanding such variations including the branching pattern of the CFA and its relationships with adjacent structures, is essential for accurate preoperative planning, minimizing surgical risks, and improving patient outcomes.

The relationship of these PFAs and their branches with the femoral nerve is noteworthy. The femoral nerve is found to be related anteriorly to the profunda arteries on both sides. In the right lower limb, the lateral PFA laterally traverses between the branches of the femoral nerve and gives branches to the quadriceps femoris muscle. This relationship has implications for surgical procedures involving the quadriceps femoris and surrounding structures.

The LCFA, originating from the lateral PFA in the right lower limb, traverses posterior to the branches of the femoral nerve. This anatomical detail is pertinent to procedures involving the blood supply to the lateral thigh region. The anterior relation of the femoral nerve to the LCFA is reported earlier by Goel et al 2015 as a ‘rare variant’. ^10^ However, Classen et al (2020) have shown in their study that the femoral nerve passed mostly anterior (46.4%) or anterior and posterior (47.8%) to the LCFA and rarely they may pass entirely posterior to the LCFA (5.8%). ^29^ The ‘obturator nerve on the left side is found situated behind the MCFA and on the right side, it is related closely to the muscular branches coming off the posteromedial PFA to the gracilis and adductor longus muscles.

The femoral and obturator nerves arise from the ventral rami of the lumbar plexus (L2-L4). The femoral nerve supplies the anterior thigh muscles and transmits sensory information to the anteromedial aspect of the thigh. The obturator nerve supplies motor and sensory signals to the inner thigh, primarily innervating the adductor muscles. Damage to either the femoral or obturator nerve can result in muscle weakness and sensory disturbances in their respective regions, with conditions such as trauma, hernias, or pelvic compression affecting these nerves.

The current investigation revealed a noteworthy difference between the right and left lower limbs. In the right limb, both the MCFA and the LCFA were found to arise from the PFA. Conversely, on the left side, the MCFA commenced from the CFA, while the LCFA stemmed from the PFA.

The present study found the ‘femoral vein’ or its tributaries traverses anteriorly to MCFA on the left and right proximal thigh region. Previous studies have reported that PFA arising medially from the femoral artery passed anterior to the femoral vein. ^8^

Comprehensive knowledge of variations in the origin, number, or course of blood vessels and their relationship with nerves is crucial for medical professionals, especially in surgery and trauma management. Understanding these connections is vital during procedures like hip surgery to minimize inadvertent damage. Surgeons must be aware of these relationships to prevent unintended nerve or vessel damage during interventions. Pathologies like aneurysms impacting arteries can affect adjacent nerves, and nerve damage may compromise blood flow by weakening the muscles, which in turn disrupts the function of the musculovenous pump, potentially leading to ischemic issues. ^30^ Radiologists interpreting imaging studies need awareness of normal anatomy for accurate diagnosis. Diseases like vasculitis can concurrently affect nerves and arteries, resulting in complex clinical presentations. Nerves depend on a consistent and sufficient blood supply to function properly, and if the arteries delivering blood to a nerve become inflamed or damaged due to vasculitis, it can result in vasculitic neuropathies. Additionally, pro-inflammatory cytokines released during vasculitis can directly target nearby nerves. ^31^ ^,^ ^32^

Overall, a deep understanding of anatomical variations in nerve-artery relationships is crucial for safe medical care in surgery, diagnostics, and clinical management.

## Conclusions

During the routine dissection of a male cadaver’s lower limb for medical students, a bilateral occurrence of double profunda femoris artery originating from the femoral artery was observed. The study delves into the nerve relationships with the profunda and its circumflex branches, emphasizing the significance of comprehending these connections for lower limb surgical and interventional procedures.

## Ethics and consent statement

The study reports findings from a single cadaver as a part of routine dissection for medical students. The cadaver used in the study was obtained through ‘Body Scientific International, LLC’. They worked with the body donation program, Medcure and Anatomical Gift Association of Illinois, where written informed consent was obtained from the donor or the donor’s legal representative prior to death on November 14, 2019. The study respects the donor’s wishes for their body to be used for educational and research purposes. The study was conducted in accordance with the principles of the Declaration of Helsinki and followed standardized protocols outlined by AQUA (Anatomical Quality Assurance) and IFAA (International Federation of Associations of Anatomists. All personal data related to the body is confidential as outlined by AQUA.

## Data Availability

No data associated with this article.
