# Supplementary material for: Case Report: Rare Bilateral Double Profunda Femoris Artery Variation: Clinical Implications and Considerations
Source: F1000Res. 2025 Sep 3;13:1528. Originally published 2024 Dec 17. [Version 3] doi: 10.12688/f1000research.151518.3 (PMC12368486; doi:10.12688/f1000research.151518.3)
Supplement: Peer review history

# Reviewer report: Reviewer response for version 3

**DOI:** 10.5256/f1000research.187425.r411470

**Published:** 2025-09-23

**Authors:** Yasith Mathangasinghe

**Affiliations:** 1 Monash University Centre for Human Anatomy Education (Ringgold ID: 589676), Clayton, Victoria, Australia

Authors have addressed my comments adequately

Are enough details provided of any physical examination and diagnostic tests, treatment given and outcomes?

No

Is the case presented with sufficient detail to be useful for other practitioners?

Yes

Is sufficient discussion included of the importance of the findings and their relevance to future understanding of disease processes, diagnosis or treatment?

Yes

Is the background of the case’s history and progression described in sufficient detail?

Yes

Reviewer Expertise:

Neuroscience, Demyelination, Oligodendrocytes, Clinical Anatomy, Medical Education

I confirm that I have read this submission and believe that I have an appropriate level of expertise to confirm that it is of an acceptable scientific standard.

# Reviewer report: Reviewer response for version 2

**DOI:** 10.5256/f1000research.179501.r393299

**Published:** 2025-08-29

**Authors:** Athikhun Suwannakhan

**Affiliations:** 1 Mahidol University, Bangkok, Thailand

The authors have revised the manuscript according to the other reviewer's comments. Embryologic description of the finding was adequately added. Anatomical descriptions of the finding are accurate and thorough. Clinical significance is well emphasized in the discussion. I have no more questions or concerns. The manuscript can be accepted for publication.

Are enough details provided of any physical examination and diagnostic tests, treatment given and outcomes?

Yes

Is the case presented with sufficient detail to be useful for other practitioners?

Yes

Is sufficient discussion included of the importance of the findings and their relevance to future understanding of disease processes, diagnosis or treatment?

Yes

Is the background of the case’s history and progression described in sufficient detail?

Yes

Reviewer Expertise:

anatomy

I confirm that I have read this submission and believe that I have an appropriate level of expertise to confirm that it is of an acceptable scientific standard.

# Response

**Published:** 2025-08-29

**Authors:** Sahar Mohsin

**Affiliations:** Anatomy, United Arab Emirates University, Al Ain, Abu Dhabi, United Arab Emirates

Response to Reviewer 4

 The authors have revised the manuscript according to the other reviewer's comments. Embryologic description of the finding was adequately added. Anatomical descriptions of the finding are accurate and thorough. Clinical significance is well emphasized in the discussion. I have no more questions or concerns. The manuscript can be accepted for publication.

 Author Response: We sincerely thank the reviewer for their thoughtful evaluation and positive feedback. We greatly appreciate your support and recommendation for acceptance.

# Reviewer report: Reviewer response for version 2

**DOI:** 10.5256/f1000research.179501.r395216

**Published:** 2025-08-20

**Authors:** Yasith Mathangasinghe

**Affiliations:** 1 Monash University Centre for Human Anatomy Education (Ringgold ID: 589676), Clayton, Victoria, Australia

General Comments

 The authors present a case report on a bilateral duplication of the profunda femoris artery (PFA) found during a cadaveric dissection. The report is well-illustrated with clear photographs and highlights an important anatomical variation with significant clinical implications. The detailed description of the arterial branching patterns and their relationships with surrounding nerves is a notable strength. The manuscript makes a useful contribution to the anatomical and clinical literature.

 Major Comments

1. The organization of the Discussion section currently feels somewhat haphazard, with key points, such as clinical significance, appearing at multiple sites. This makes the narrative difficult to follow. I recommend restructuring the discussion to follow a more logical sequence, which would significantly improve its impact. A suggested structure could be:
  - Embryological Basis & Prevalence: Begin with the embryological development that can lead to this variation (which the authors have added in version 2) and a review of the prevalence from previous reports.
  - Consolidated Clinical Significance: Create a single, robust section dedicated to the clinical implications. While the authors mention orthopaedic surgery and interventional radiology, this could be greatly expanded. I suggest elaborating on how this specific variation could impact:
    - Vascular Surgery: The presence of a second PFA could be missed during angiography, leading to failed bypass grafts, incomplete aneurysm embolization or repair, or misinterpretation of bleeding sources.
    - Reconstructive Surgery: Unrecognized variations can compromise the viability of essential tissue flaps that rely on perforators from the PFA system, such as the anterolateral thigh (ALT) flap, by creating an unpredictable blood supply.
    - Trauma Surgery: In cases of pelvic or femoral fractures, attempts to control hemorrhage could fail if only one of two major vessels is ligated or embolized.
2. The manuscript states that a double PFA may have an effect on femoral catheterization. This claim requires more substantiation. Standard femoral artery access is typically gained in the common femoral artery (CFA), proximal to the PFA bifurcation. It is not immediately clear how a duplicated PFA, originating distal to the typical puncture site, would directly complicate initial access. Could the authors please elaborate on the specific scenario where this becomes a clinical issue (e.g., during the distal advancement of sheaths, risk of dissection) and provide a citation to support this statement? If no direct evidence is available, it may be better left off.
3. In the Background section, the authors describe the duplication of the PFA as "extremely rare," citing Tsoucalas et al. While I appreciate the reference, I would caution against this term and suggest a more quantitatively precise description. It would be more meaningful for the reader to use the term "uncommon" and state the known prevalence directly (e.g., "with a reported prevalence of <1%"). This provides clearer context. As a reference point, the NIH often defines a rare condition as affecting fewer than 1 in 2,000 people, which helps contextualize such terminology. Using specific prevalence data is more scientifically rigorous than relying on a qualitative descriptor.

Minor Comments

1. The manuscript could be made more concise by addressing a few redundant statements. For example, the role of the PFA in supplying the medial and posterior thigh compartments is mentioned multiple times across the Background and Discussion sections. Consolidating this information would improve the flow and readability of the text.
2. The abstract mentions, "...The LCFA stems from the upper lateral PFA..." The term "upper lateral PFA" is only clear after reading the full results. Consider rephrasing for clarity in the abstract, perhaps by referring to it as one of the two duplicated arteries.
3. The first sentence of the Background states the femoral artery "originates" from the external iliac artery. It is more accurate to state that it is the continuation of the external iliac artery distal to the inguinal ligament.
4. The use of inverted commas around anatomical terms (e.g., ‘femoral vein’, ‘pectineus’) is unnecessary and not standard practice. These can be removed throughout the manuscript.

Are enough details provided of any physical examination and diagnostic tests, treatment given and outcomes?

No

Is the case presented with sufficient detail to be useful for other practitioners?

Yes

Is sufficient discussion included of the importance of the findings and their relevance to future understanding of disease processes, diagnosis or treatment?

Yes

Is the background of the case’s history and progression described in sufficient detail?

Yes

Reviewer Expertise:

Neuroscience, Demyelination, Oligodendrocytes, Clinical Anatomy, Medical Education

I confirm that I have read this submission and believe that I have an appropriate level of expertise to confirm that it is of an acceptable scientific standard, however I have significant reservations, as outlined above.

# Response

**Published:** 2025-08-28

**Authors:** Sahar Mohsin

**Affiliations:** Anatomy, United Arab Emirates University, Al Ain, Abu Dhabi, United Arab Emirates

**Response to Reviewer 3 Comments**

 The authors present a case report on a bilateral duplication of the profunda femoris artery (PFA) found during a cadaveric dissection. The report is well-illustrated with clear photographs and highlights an important anatomical variation with significant clinical implications. The detailed description of the arterial branching patterns and their relationships with surrounding nerves is a notable strength. The manuscript makes a useful contribution to the anatomical and clinical literature.

 Major Comments

1. The organization of the Discussion section currently feels somewhat haphazard, with key points, such as clinical significance, appearing at multiple sites. This makes the narrative difficult to follow. I recommend restructuring the discussion to follow a more logical sequence, which would significantly improve its impact. A suggested structure could be:
  - Embryological Basis & Prevalence: Begin with the embryological development that can lead to this variation (which the authors have added in version 2) and a review of the prevalence from previous reports.
  - Consolidated Clinical Significance: Create a single, robust section dedicated to the clinical implications. While the authors mention orthopaedic surgery and interventional radiology, this could be greatly expanded. I suggest elaborating on how this specific variation could impact:
    - Vascular Surgery: The presence of a second PFA could be missed during angiography, leading to failed bypass grafts, incomplete aneurysm embolization or repair, or misinterpretation of bleeding sources.
    - Reconstructive Surgery: Unrecognized variations can compromise the viability of essential tissue flaps that rely on perforators from the PFA system, such as the anterolateral thigh (ALT) flap, by creating an unpredictable blood supply.
    - Trauma Surgery: In cases of pelvic or femoral fractures, attempts to control hemorrhage could fail if only one of two major vessels is ligated or embolized.

**Author response**: We sincerely thank the reviewer for the constructive comments and valuable suggestions. As advised, the discussion section has been thoroughly restructured to improve clarity and logical flow. The revised version now begins with the embryological background, followed by a review of the prevalence from previous reports. The clinical significance of the variation has been elaborated in a structured manner under separate sub-sections for vascular surgery, orthopedic and trauma surgery, and reconstructive surgery, with specific emphasis on how this rare bilateral double PFA variation may impact each specialty. Finally, the comparative literature and novel findings from the present case are highlighted, followed by the broader clinical relevance.

 We appreciate the reviewer’s input, which has significantly improved the organization and impact of the discussion.

 2. The manuscript states that a double PFA may have an effect on femoral catheterization. This claim requires more substantiation. Standard femoral artery access is typically gained in the common femoral artery (CFA), proximal to the PFA bifurcation. It is not immediately clear how a duplicated PFA, originating distal to the typical puncture site, would directly complicate initial access. Could the authors please elaborate on the specific scenario where this becomes a clinical issue (e.g., during the distal advancement of sheaths, risk of dissection) and provide a citation to support this statement? If no direct evidence is available, it may be better left off

 . **Author response**: We have deleted ‘femoral catheterization’ from the text as suggested.

 3.In the Background section, the authors describe the duplication of the PFA as "extremely rare," citing Tsoucalas et al. While I appreciate the reference, I would caution against this term and suggest a more quantitatively precise description. It would be more meaningful for the reader to use the term "uncommon" and state the known prevalence directly (e.g., "with a reported prevalence of <1%"). This provides clearer context. As a reference point, the NIH often defines a rare condition as affecting fewer than 1 in 2,000 people, which helps contextualize such terminology. Using specific prevalence data is more scientifically rigorous than relying on a qualitative descriptor.

**Author response**: We have revised the Background section to replace the qualitative term “extremely rare” with a more precise description. The duplication of the profunda femoris artery is now described as **“** **uncommon, with a reported prevalence of <1%”**, providing quantitative context for the reader. This adjustment aligns with the reviewer’s recommendation to use scientifically rigorous prevalence data rather than a subjective descriptor.

 Minor Comments

1. The manuscript could be made more concise by addressing a few redundant statements. For example, the role of the PFA in supplying the medial and posterior thigh compartments is mentioned multiple times across the Background and Discussion sections. Consolidating this information would improve the flow and readability of the text.

**Author response**: We thank the reviewer for the suggestion. Upon review, we did not find redundancy regarding the PFA supplying the medial and posterior thigh compartments in the Discussion section. References to these regions appear only in the Background to provide context.

 2. The abstract mentions, "...The LCFA stems from the upper lateral PFA..." The term "upper lateral PFA" is only clear after reading the full results. Consider rephrasing for clarity in the abstract, perhaps by referring to it as one of the two duplicated arteries.

**Author response**: We thank the reviewer for highlighting the ambiguity in the abstract. The phrase “upper lateral PFA” has been revised to **“upper lateral branch of the duplicated PFA”** to clarify that the LCFA arises from one of the two PFAs without requiring reference to the full results section.

 3. The first sentence of the Background states the femoral artery "originates" from the external iliac artery. It is more accurate to state that it is the continuation of the external iliac artery distal to the inguinal ligament

**Author response** : We thank the reviewer for the comment. The text has been revised for clarity.

 The original sentence:

 “Originating from the common iliac artery in front of the sacroiliac joints at the level of L5-S1, the femoral artery is the continuation of the external iliac artery. It enters the lower limb region behind the inguinal ligament, located halfway between the anterior superior iliac spine and the pubic symphysis.

 has been updated to:

 “The femoral artery is the continuation of the external iliac artery distal to the inguinal ligament, which is located approximately halfway between the anterior superior iliac spine and the pubic symphysis. The common iliac artery divides into the internal and external iliac arteries at the level of L5–S1.”

  

1. The use of inverted commas around anatomical terms (e.g., ‘femoral vein’, ‘pectineus’) is unnecessary and not standard practice. These can be removed throughout the manuscript.

**Author response**: Done. Thanks for bring it to our attention.

- **Is the background of the case’s history and progression described in sufficient detail?**

Yes

- **Are enough details provided of any physical examination and diagnostic tests, treatment given and outcomes?**

No

- **Is sufficient discussion included of the importance of the findings and their relevance to future understanding of disease processes, diagnosis or treatment?**

Yes

- **Is the case presented with sufficient detail to be useful for other practitioners?**

Yes

# Response

**Published:** 2025-08-31

**Authors:** Soumya Vinod

**Affiliations:** Basic Sciences, United Arab Emirates University, Abu Dhabi, United Arab Emirates

1. Some sections require minor restructuring to improve overall flow. Revise these sections to ensure logical progression of ideas. While the study contributes to existing literature, a clearer statement outlining its unique contributions is needed. I suggest the authors to add a paragraph in the discussion to highlight novel aspects and how this finding is novel compared to previous studies.

**Reply:** The following paragraph will be added in the discussion (Page 11. Line 173, Second paragraph)

 The novelty of the present study was morphometric and volumetric measurement and analysis of the three major paranasal sinuses altogether (frontal, maxillary and sphenoidal), with the help of high-resolution multidetector CT scans when we began the study.  But in recent years several studies have been conducted on the same concept. Therefore, at present the results of our study add to the existing data and might help in future studies.

The following paragraph will also be added in Page 11. Line 197

 The agenesis of frontal sinus we observed in our study and the finding that the age of the individual does not correlate with the sinus measurements significantly (19-73years) is comparatively rare and it might help in forensic analysis. Additionally, the usage of contrast-based volume tracing might help in 3D reconstruction improve the strategies for accuracy of measurement and may serve as a template for future forensic imaging protocols.

 2. Enhance clarity by including a brief overview of the paranasal sinus anatomy and a short section on their embryological development in the introduction.

**Reply:** The following paragraph will be added to the Introduction in page 3, line 21

 Paranasal sinuses are air filled spaces communicated with the nasal cavity and they are present within the craniofacial bones like frontal, ethmoid, sphenoid and maxilla (Standring, 2021). Their development begins during intrauterine life through a process known as pneumatization (Fahrioglu et al, 2023). They begin to develop in between the 3 ^rd^ to 4 ^th^ month of gestation, with maxillary and then ethmoidal sinus being the first to develop (Lee et al, 2022; Moore et al, 2016). Sphenoidal sinus begins to develop around 4 ^th^ month, and the maxillary sinus is the last to develop. Maxillary sinus is usually primitive at birth and continues to expand around 5 to 6 years postnatally and attains maximum volume around 18-20 years (Suman et al, 2016). Structurally these sinuses stay stable throughout adulthood except when affected by trauma, pathological consequences or surgeries and this feature enables their use in forensic applications (Suman et al, 2016). 

- Standring S. Gray’s Anatomy: The anatomical basis for clinical practice. 42 ^nd^ Ed. 2021. Chapter 39. Pp 686-701,33 546-55717 273-291.
- Fahrioglu LS, VanKampen N, Andaloro C. Anatomy, Head and Neck, Sinus Function and Development. Updated 2023 Jan 30. In: StatPearls [Internet]. Treasure Island (FL): StatPearls Publishing; 2025 Jan-. Available from: https://www.ncbi.nlm.nih.gov/books/NBK532926/
- Lee S, Fernandez J, Mirjalili SA, Kirkpatrick J. Pediatric paranasal sinuses-Development, growth, pathology, & functional endoscopic sinus surgery. Clin Anat. 2022 Sep;35(6):745-761. doi: 10.1002/ca.23888. 
- Moore KL, Persaud TVN, Torchia MG. The Developing Human: Clinically Oriented Embryology. 10th ed. Philadelphia: Elsevier; 2016.
- **Suman JL, Jaisanghar N, Elangovan S, et al.** Configuration of frontal sinuses: A forensic perspective. *J Pharm* *Bioallied Sci.* 2016;8(Suppl 1):S90–S95. https://doi.org/10.4103/0975-7406.192031

3. Include a section discussing the limitations of using CT scans, such as costs and equipment accessibility, and address potential pathologies that may affect sinus measurements.

**Reply:** The following paragraph will be added at the end of the discussion in page 13, line 226

 Though the fact that CT provides high resolution images they are associated with certain limitations like being expensive, low resource especially in rural settings (Power et al, 2016). Moreover, the risk of ionizing radiation exposure, in case of multiple scan requirements in particular (Fazlalizadeh et al, 2024). Though these are minimal limitations it might affect the large-scale forensic applications. Likewise, chronic sinusitis or certain other pathologies of the sinus might alter the morphology of the affected sinus and negatively influence the accuracy of measurements and interpretation.

- Power SP, Moloney F, Twomey M, James K, O'Connor OJ, Maher MM. Computed tomography and patient risk: Facts, perceptions and uncertainties. World J Radiol. 2016 Dec 28;8(12):902-915. doi: 10.4329/wjr.v8.i12.902. 
- Fazlalizadeh H, Khan MS, Fox ER, et al. Closing the Last Mile Gap in Access to Multimodality Imaging in Rural Settings: Design of the Imaging Core of the Risk Underlying Rural Areas Longitudinal Study. Circ Cardiovasc Imaging. 2024 Feb;17(2):e015496. doi: 10.1161/CIRCIMAGING.123.015496. 

 

 4. The discussion would benefit from a deeper comparison with findings from other populations and an elaboration on clinical implications for forensic identification.

**Reply:** We have compared the dimensions in the 5 ^th^ paragraph under discussion (line 186-195).

However, the following paragraph has been added to the manuscript (page 11, line 203)

**In clinical and forensic practice, such morphometric data can aid in sex estimation when traditional identifiers are unavailable—especially in cases involving severe trauma, explosions, or natural disasters where only fragmented skull components remain.**

 5. Include a table in the results section displaying the range of measured dimensions and volumes for a comprehensive overview of the data.

**Reply:** We appreciate the suggestion to include a table displaying the range of measured dimensions and volumes. However, due to the manuscript's total limit of seven figures and tables, we were unable to include this information in a separate table.

# Reviewer report: Reviewer response for version 2

**DOI:** 10.5256/f1000research.179501.r379771

**Published:** 2025-05-03

**Authors:** Cagatay Barut

**Affiliations:** 1 Istanbul Medeniyet University, Istanbul, Turkey

The authors have made the suggested revisions, and in its current form, the manuscript is acceptable for indexing.

Are enough details provided of any physical examination and diagnostic tests, treatment given and outcomes?

Partly

Is the case presented with sufficient detail to be useful for other practitioners?

Yes

Is sufficient discussion included of the importance of the findings and their relevance to future understanding of disease processes, diagnosis or treatment?

Partly

Is the background of the case’s history and progression described in sufficient detail?

Yes

Reviewer Expertise:

Clinical anatomy, anatomical variaitons, radiologic anatomy

I confirm that I have read this submission and believe that I have an appropriate level of expertise to confirm that it is of an acceptable scientific standard.

# Reviewer report: Reviewer response for version 1

**DOI:** 10.5256/f1000research.166167.r363314

**Published:** 2025-03-04

**Authors:** Ivan James Prithishkumar

**Affiliations:** 1 Mohammed Bin Rashid University of Medicine and Health Sciences, Dubai, United Arab Emirates

Abstract:

1. What do you mean by upper PFA in the sentence, ‘…The LCFA stems from the upper PFA…..’

Background:

 1. The femoral artery is does not originate from the 'external iliac artery'. It is a continuation of the 'external iliac artery'. Please rephrase.

 2. Replace '...opening in the adductor magnus' with '

 3. Remove the inverted comma in the sentence...and the ‘medial’ and ‘posterior’ compartments of the thigh...

 4. What is LCFA? Write the full form.

 5. Remove the sentence https://en.wikipedia.org/wiki/Lateral_circumflex_femoral_artery .from the main text. Please do not reference wikipedia.

 Methods:

 1. Did you dissect one cadaver or many cadavers? It is not very clear here. How many cadavers or lower limbs were dissected.

- Please mention that these cadavers were donated through the body donation program for teaching and research purpose in the methodology section also.
- Do you have more information about the cadaver such as age, race, height, or cause of death. It should be available in the body donation documents. Please include if available.

3. Cunninghams Manual of Practical Anatomy (Mention the full name of the book) and reference it.

 4. Please remove the ‘…..’ inverted commas from the sentence, “It was observed that the ‘femoral artery’ follows…” Also, please remove the commas from all other words throughout the manuscript. There is no need to put the inverted commas.

 Discussion:

1. You have mentioned that the variation can pose crucial implications for medical professionals. What procedures can be implicated or complicated due to this variation. List a few.
2. Does nerve damage compromise blood supply? How and Why? Provide references to support the text.
3. How and why does vasculitis affect the adjacent nerves? Justify using references

Acknowledgments:

1. Replace the phrase “…for taking care’ of the cadavers to ‘preservation and maintenance of the cadavers’.  

References:

1. Include references from recent publications if available. Maybe last 3 years.

Are enough details provided of any physical examination and diagnostic tests, treatment given and outcomes?

Yes

Is the case presented with sufficient detail to be useful for other practitioners?

Yes

Is sufficient discussion included of the importance of the findings and their relevance to future understanding of disease processes, diagnosis or treatment?

Yes

Is the background of the case’s history and progression described in sufficient detail?

Yes

Reviewer Expertise:

Clinical Anatomy

I confirm that I have read this submission and believe that I have an appropriate level of expertise to confirm that it is of an acceptable scientific standard, however I have significant reservations, as outlined above.

# Response

**Published:** 2025-04-11

**Authors:** Sahar Mohsin

**Affiliations:** Anatomy, United Arab Emirates University, Al Ain, Abu Dhabi, United Arab Emirates

Response to Reviewer 2

 Abstract:

 Thank you for taking the time to review this case report and for providing your valuable feedback. We greatly appreciate your insights and thoughtful comments. In response, we have revised the manuscript according to your suggestions and are grateful for your constructive input.

1. What do you mean by upper PFA in the sentence, ‘…The LCFA stems from the upper PFA…..’

**Thank you for your valuable feedback. In our sentence, “The LCFA stems from the upper PFA…”, the term "upper PFA" refers to the first PFA (Profunda Femoris Artery), which originates laterally from the CFA, approximately 2.5 cm from the inguinal ligament.**

 As described later in the manuscript, **‘on page 6 under section heading** ‘Origin of double profunda femoral arteries in the left thigh region of the lower limb” ‘The first PFA commences laterally from the CFA, approximately 2.5 centimetres from the inguinal ligament, while the second PFA originates below the first one at a distance of 5 centimetres from the inguinal ligament and is posterior to the CFA at its commencement and later traverses laterally to the ‘femoral artery’. The lower posterior PFA is bigger than the upper lateral PFA.

**To maintain clarity and consistency, we have revised the term "upper PFA" to "upper lateral PFA”  in the abstract and in the manuscript**

**To improve the clarity in the abstract, we have rephrased the sentence as ‘The LCFA stems from the upper lateral PFA, while the medial MCFA arises from the CFA.”**

 Background:

 1. The femoral artery is does not originate from the 'external iliac artery'. It is a continuation of the 'external iliac artery'. Please rephrase.

**Done**

 2. Replace '...opening in the adductor magnus' with '

**Commas are deleted from the adductor magnus and the popliteal artery.**

 3. Remove the inverted comma in the sentence...and the ‘medial’ and ‘posterior’ compartments of the thigh...

**Done**

 4. What is LCFA? Write the full form.

**Done for both LCFA and MCFA**

 5. Remove the sentence https://en.wikipedia.org/wiki/Lateral_circumflex_femoral_artery .from the main text. Please do not reference wikipedia.

**Done**

 Methods:

1. Did you dissect one cadaver or many cadavers? It is not very clear here. How many cadavers or lower limbs were dissected.

**This case report is based on the findings from one male cadaver. We have mentioned it in the ethics and consent statement, and now added to the methodology section as well.**

  

1. Please mention that these cadavers were donated through the body donation program for teaching and research purpose in the methodology section also.

**Done**

1. Do you have more information about the cadaver such as age, race, height, or cause of death. It should be available in the body donation documents. Please include if available.

**We have shared the patient consent form with the journal but it is not possible to publish any personal data as per ethical guidelines.**

  

1. Cunninghams Manual of Practical Anatomy (Mention the full name of the book) and reference it.

**Done. Thanks for bringing to our attention.**

  

1. Please remove the ‘…..’ inverted commas from the sentence, “It was observed that the ‘femoral artery’ follows…” Also, please remove the commas from all other words throughout the manuscript. There is no need to put the inverted commas.

**Done.**

 Discussion:

1. You have mentioned that the variation can pose crucial implications for medical professionals. What procedures can be implicated or complicated due to this variation. List a few.

**We rephrased the paragraph and added some surgical procedures as requested.**

1. Does nerve damage compromise blood supply? How and Why? Provide references to support the text.

We expanded the following text to further explain the statement ‘Pathologies like aneurysms impacting arteries can affect adjacent nerves, and nerve damage can impair blood flow **by weakening the muscles, which in turn disrupts the function of the musculovenous pump, **potentially leading to ischemic issues ^”^.

 We added the following reference to support this statement.

 Casey DP, Hart EC. Cardiovascular function in humans during exercise: role of the muscle pump. J Physiol. 2008 Nov 1;586(21):5045-6. doi: 10.1113/jphysiol.2008.162123.

1. How and why does vasculitis affect the adjacent nerves? Justify using references

**We have expanded the text to explain the concept by adding this statement**

**‘Nerves depend on a consistent and sufficient blood supply to function properly, and if the arteries delivering blood to a nerve become inflamed or damaged due to vasculitis, it can result in vasculitic neuropathies. Additionally, pro-inflammatory cytokines released during vasculitis can directly target nerves.**

**We have added two references that explain the mechanisms in detail:-**

**Landman, A.; Levine, M.; De León, A. M. Vasculitic Neuropathies. *Current Treatment Options in Neurology* 2024, *26* (3), 67–77. https://doi.org/10.1007/s11940-024-00782-x.**

**Gwathmey, K. G.; Burns, T. M.; Collins, M. P.; Dyck, P. J. B. Vasculitic Neuropathies. *The Lancet Neurology* 2014; *13* (1), 67–82. https://doi.org/10.1016/S1474-4422(13)70236-9.**

**Acknowledgments:**

1. Replace the phrase “…for taking care’ of the cadavers to ‘preservation and maintenance of the cadavers’.  

**Done**

**References:**

1. Include references from recent publications if available. Maybe last 3 years.

**We have included recent references from 2021 and 2024; however, due to the rarity of this anomaly, there are limited recent publications available**

# Reviewer report: Reviewer response for version 1

**DOI:** 10.5256/f1000research.166167.r350366

**Published:** 2025-01-06

**Authors:** Cagatay Barut

**Affiliations:** 1 Istanbul Medeniyet University, Istanbul, Turkey

The authors presented a bilateral double profunda femoris artery, an important variation. I highly recommend that the authors add a detailed embryology of these vessels to better understand such variations. Embryology should not only cover the embryology in the textbooks but the authors should focus on the literature and present a detailed embryology of the femoral artery and the formation of the profunda femoris artery. Thus the background regarding this variation will be explained using embryology. Several examples of literature are listed below:

Are enough details provided of any physical examination and diagnostic tests, treatment given and outcomes?

Partly

Is the case presented with sufficient detail to be useful for other practitioners?

Yes

Is sufficient discussion included of the importance of the findings and their relevance to future understanding of disease processes, diagnosis or treatment?

Partly

Is the background of the case’s history and progression described in sufficient detail?

Yes

Reviewer Expertise:

Clinical anatomy, anatomical variaitons, radiologic anatomy

I confirm that I have read this submission and believe that I have an appropriate level of expertise to state that I do not consider it to be of an acceptable scientific standard, for reasons outlined above.

## References

[1] : Embryology of the lower limb demonstrates that congenital absent fibula is a radiologic misnomer. *Anat Rec (Hoboken)* .2022;305(1) : 10.1002/ar.24628 8-17 10.1002/ar.24628 33773063

[2] : Arteries of the Lower Limb-Embryology, Variations, and Clinical Significance. *Can Assoc Radiol J* .2022;73(1) : 10.1177/08465371211003860 259-270 10.1177/08465371211003860 33886403

[3] Reference source

# Response

**Published:** 2025-01-07

**Authors:** Sahar Mohsin

**Affiliations:** Anatomy, United Arab Emirates University, Al Ain, Abu Dhabi, United Arab Emirates

Thank you for your thoughtful and constructive comments on our manuscript titled "Case Report: Rare Bilateral Double Profunda Femoris Artery Variation: Clinical Implications and Considerations."

 We sincerely appreciate the time you took to review our work and the valuable insights you provided. 

 We understand the importance of embryological context in understanding anatomical variations, and we agree that explaining the embryology behind the formation of the femoral artery and profunda femoris artery would enrich the manuscript. The main objective of the case report is to highlight the rare anatomical variation and its clinical implications, especially for surgical and diagnostic procedures, we prioritized its practical and clinical relevance over embryological discussion due to the concise format of a case report. 

 In response to the comment, we propose adding a concise section on the embryological development of these arteries, referencing pertinent literature to bridge the anatomical findings with developmental origins. This would allow us to bridge the gap between the anatomical findings and their developmental origins without overshadowing the main clinical emphasis of the case report. 

 We hope this approach addresses your concern while preserving the integrity and purpose of our case report. We would be grateful for your reconsideration of the manuscript based on these adjustments. Once again, thank you for your valuable feedback. We look forward to your thoughts on our proposed revisions.

# Response

**Published:** 2025-04-11

**Authors:** Sahar Mohsin

**Affiliations:** Anatomy, United Arab Emirates University, Al Ain, Abu Dhabi, United Arab Emirates

**Response to Reviewer 1**

**Comment**

 The authors presented a bilateral double profunda femoris artery, an important variation. I highly recommend that the authors add a detailed embryology of these vessels to better understand such variations. Embryology should not only cover the embryology in the textbooks but the authors should focus on the literature and present a detailed embryology of the femoral artery and the formation of the profunda femoris artery. Thus the background regarding this variation will be explained using embryology. Several examples of literature are listed below:

 References

 1. Hootnick DR, Levinsohn EM: Embryology of the lower limb demonstrates that congenital absent fibula is a radiologic misnomer. *Anat Rec (Hoboken)*. 2022; 305 (1): 8-17 PubMed Abstract | Publisher Full Text

 2. Qazi E, Wilting J, Patel NR, Alenezi AO, et al.: Arteries of the Lower Limb-Embryology, Variations, and Clinical Significance. *Can Assoc Radiol J*. 2022; 73 (1): 259-270 PubMed Abstract |

**Response**

 Thank you for your thoughtful and constructive comments on our manuscript titled "Case Report: Rare Bilateral Double Profunda Femoris Artery Variation: Clinical Implications and Considerations." We sincerely appreciate the time you took to review our work and the valuable insights you provided.

 We understand the importance of embryological context in understanding anatomical variations, and we agree that explaining the embryology behind the formation of the femoral artery and profunda femoris artery would enrich the manuscript. The main objective of the case report is to highlight the rare anatomical variation and its clinical implications, especially for surgical and diagnostic procedures, we prioritized its practical and clinical relevance over embryological discussion due to the concise format of a case report.

 In response to the comment, we added the following paragraph in the discussion section:-

 Blood vessel formation involves vasculogenesis and angiogenesis. ^14,15,16^ The molecular control of vascular development is complex, involving growth factors and inhibitors. Vascular remodeling requires vessel sprouting, intussusceptive growth, and regulation by hemodynamic, growth, and inhibitory factors, along with tissue environment. Variations in these processes, due to genetic factors, failed regulation, or environmental influences (e.g., hypoxemia), can lead to vessel abnormalities like duplication, hypoplasia, or aplasia. ^17-19^

 The common dorsal aorta forms intersegmental arteries, with the external iliac artery developing from the fifth lumbar intersegmental artery. ^20^ The primary (axial)artery of the lower limb called the sciatic artery emerges from the common iliac artery, develops from the umbilical artery of the dorsal aorta. ^14^ It extends from the medial thigh to the lateral leg and forms a plantar plexus in the sole. External iliac/femoral artery penetrate into the lower limb after the axial artery, it transiently joins the axial/sciatic artery near the knee to form the popliteal artery. ^20^  Between the 6 mm and 33 mm stages, the sciatic artery regresses and the dorsal thigh is primarily supplied by the profunda femoris, which branches off the femoral artery. ^21,22^ However, persistence of the sciatic artery as the principal blood supply to the lower limb in adulthood is an uncommon vascular abnormality with potential surgical implications. ^23^ The arterial system of the lower limb is fully formed by the 33-mm stage of embryonic development, the embryonic femoral artery at first comprises several vascular channels, which gradually merge to give rise to the profunda femoral arteries. If these channels fail to fuse properly, a duplicated profunda femoris artery may result. ^24^ The vasculature adopts its adult configuration by the 8th week of embryonic development. Other rare variations include an absent deep femoral artery and lateral and medial circumflex arteries originating from the common or superficial femoral artery instead of the deep femoral artery. ^25,26^

 Anatomical variations of the femoral artery are uncommon and may include the absence of the profunda femoris artery, a persistent sciatic artery, or duplication of the superficial femoral artery. Most of these variations result from the persistence of primitive arterial segments, abnormal fusions, or segmental hypoplasia or aplasia. ^15^, ^19^

 We have added the following references for the recommended text

  

1. Qazi E, Wilting J, Patel NR, Alenezi AO, Kennedy SA, Tan KT, Jaberi A, Mafeld S. Arteries of the Lower Limb-Embryology, Variations, and Clinical Significance. *Can Assoc Radiol J.* 2022 Feb;73(1):259-270. doi: 10.1177/08465371211003860. Epub 2021 Apr 22.
2. Hootnick DR, Levinsohn EM. Embryology of the lower limb demonstrates that congenital absent fibula is a radiologic misnomer. Anat Rec (Hoboken). 2022 ;305(1):8-17. doi: 10.1002/ar.24628
3. Vargesson N, Hootnick DR. Arterial dysgenesis and limb defects: Clinical and experimental examples. Reprod Toxicol. 2017 ;70:21-29. doi: 10.1016/j.reprotox.2016.10.005
4. DeSesso JM. Vascular ontogeny within selected thoracoabdominal organs and the limbs. *Reprod Toxicol*. 2017;70:3–20.
5. Foster TR, Chin JA, Brownson KE, Bai H, Dardik A. Embryological Basis for Vascular Anomalies. Human Fetal Growth and Development. Springer; 2016: 349–358.
6. Kil S-W, Jung G-S. Anatomical variations of the popliteal artery and its tibial branches: analysis in 1242 extremities. *Cardiovasc Intervent Radiol*. 2009;32(2):233–240
7. Khalid N, Bordoni B. Embryology, Great Vessel. 2023 May 1. In: StatPearls [Internet]. Treasure Island (FL): StatPearls Publishing; 2025.
8. Yu SH, Hwang JH, Kim JH, Park S, Lee KH, Choi ST. Duplication of superficial femoral artery: imaging findings and literature review. *BMC Med Imaging*. 2020 ;20(1):99. doi: 10.1186/s12880-020-00500-4.
9. Hapugoda S, Hsu CC, Kwan GN, Watkins TW, Rophael JA. Duplication of the superficial femoral artery: comprehensive review of imaging literature and insight into embryology. *Acta Radiol Open*. 2016 Jul 26;5(7):2058460116659098. doi: 10.1177/2058460116659098.
10. Brantley SK, Rigdon EE, Raju S. Persistent sciatic artery: embryology, pathology, and treatment. *J Vasc Surg*. 1993 Aug;18(2):242-8.

 

1. Kantarci F, Mihmanli I, Aksoy H, Barutca H, Gurses B, Kaynak K. Duplication of the superficial femoral artery diagnosed primarily on the basis of color Doppler ultrasonography. *J Ultrasound Med*. 2003 Jun;22(6):641-3. doi: 10.7863/jum.2003.22.6.641.
2. Swift H, Bordoni B. Anatomy, Bony Pelvis and Lower Limb: Femoral Artery. 2023 Nov 29. In: StatPearls [Internet]. Treasure Island (FL): StatPearls Publishing; 2025 Jan.

We hope this approach addresses your concern while preserving the integrity and purpose of our case report. We would be grateful for your reconsideration of the manuscript based on these adjustments. Once again, thank you for your valuable feedback. We look forward to your thoughts on our proposed revisions.
